# Metagenomic analysis reveals the shared and distinct features of the soil resistome across tundra, temperate prairie, and tropical ecosystems

**DOI:** 10.1186/s40168-021-01047-4

**Published:** 2021-05-14

**Authors:** Xun Qian, Santosh Gunturu, Jiarong Guo, Benli Chai, James R. Cole, Jie Gu, James M. Tiedje

**Affiliations:** 1 Interdisciplinary Research Center for Soil Microbial Ecology and Land Sustainable Productivity in Dry Areas, Northwest A&F University, Yangling, 712100 Shaanxi China; 2 Center for Microbial Ecology, Michigan State University, East Lansing, MI 48824 USA; 3 College of Natural Resources and Environment, Northwest A&F University, Yangling, 712100 Shaanxi China

**Keywords:** Soil resistome, Geographical distribution, Background ARG, Clinical ARG, Anthropogenic impact

## Abstract

**Background:**

Soil is an important reservoir of antibiotic resistance genes (ARGs), but their potential risk in different ecosystems as well as response to anthropogenic land use change is unknown. We used a metagenomic approach and datasets with well-characterized metadata to investigate ARG types and amounts in soil DNA of three native ecosystems: Alaskan tundra, US Midwestern prairie, and Amazon rainforest, as well as the effect of conversion of the latter two to agriculture and pasture, respectively.

**Results:**

High diversity (242 ARG subtypes) and abundance (0.184–0.242 ARG copies per 16S rRNA gene copy) were observed irrespective of ecosystem, with multidrug resistance and efflux pump the dominant class and mechanism. Ten regulatory genes were identified and they accounted for 13–35% of resistome abundances in soils, among them *arlR*, *cpxR*, *ompR*, *vanR*, and *vanS* were dominant and observed in all studied soils. We identified 55 non-regulatory ARGs shared by all 26 soil metagenomes of the three ecosystems, which accounted for more than 81% of non-regulatory resistome abundance. Proteobacteria, Firmicutes, and Actinobacteria were primary ARG hosts, 7 of 10 most abundant ARGs were found in all of them. No significant differences in both ARG diversity and abundance were observed between native prairie soil and adjacent long-term cultivated agriculture soil. We chose 12 clinically important ARGs to evaluate at the sequence level and found them to be distinct from those in human pathogens, and when assembled they were even more dissimilar. Significant correlation was found between bacterial community structure and resistome profile, suggesting that variance in resistome profile was mainly driven by the bacterial community composition.

**Conclusions:**

Our results identify candidate background ARGs (shared in all 26 soils), classify ARG hosts, quantify resistance classes, and provide quantitative and sequence information suggestive of very low risk but also revealing resistance gene variants that might emerge in the future.

Video abstract

**Supplementary Information:**

The online version contains supplementary material available at 10.1186/s40168-021-01047-4.

## Background

Antibiotic resistance is a global threat to public health, to which an estimated 700,000 yearly deaths are attributed and is predicted to cause 10 million deaths by 2050 if unchecked [1]. Soils are likely the most significant antibiotic resistance gene (ARG) reservoirs, and large amounts and diversities of ARGs have been found in soils throughout the world [2–4], including some in Antarctic surface soils [5]. Many clinically relevant ARGs originated from the soil resistome via horizontal gene transfer [6, 7]. Further studies demonstrate that some anthropogenic activities significantly enrich the abundance of indigenous ARGs in soils [8–10]. Understanding soil resistomes at a broad geographic scale and across major ecosystems, especially in native soils which have not been exposed to anthropogenic activities, can help better define the background levels and types of ARGs, which is essential for assessing the potential risk of new human activities.

Different land use practices can significantly alter the soil physicochemical as well as biological properties. Conversion of native soil to crop cultivation has been one of the most common anthropogenic land use changes. For example, 100 years of continuous cultivation significantly changed microbial diversity and structure in a consistent though not major way in North American Midwest prairie soils [11]. Further, the conversion of Amazon rainforest to cattle pasture is another land use change that has expanded in the tropics [12], and has led to homogenization of microbial communities [13]. Since land use change has been and continues to be the most extensive alteration of the terrestrial environment, its impact on the soil resistome is important to understand.

Most of our knowledge of ARGs in soils has come from targeting those genes by real-time quantitative PCR (RT-qPCR) including highly parallel qPCR platforms [14–16]. While qPCR is more sensitive, it is limited to the known genes and the specificities imposed by primers. Metagenomics (shotgun sequencing) is now affordable and provides a more comprehensive overview of environmental resistomes [17, 18]. Furthermore, new ARG bioinformatic analysis tools are available to efficiently analyze this large amount of data [19, 20]. Since different ARGs have different levels of risk [21], it is necessary to categorize ARGs by their functional roles, the necessary components for their resistance function, and assess their potential risk separately rather than merely the abundance of total ARGs.

With the increasing availablity of metagenomic data in public databases, a few studies have used that data to provide a global view of the soil resistome [4, 22–25]. While these studies provide a useful overview, they lose resolution on the effect of different ecologies, which is important to understand soil community assemblies and their relatiohships to its resistome. Here, by using a hierarchical structured (ARGs type-subtype-reference sequence) database and ARGs-OAP pipeline [26, 27], we investigated impacts of land use change on the soil resistome with soils matched for edaphic traits and land form for two major ecosystem types, i.e., U.S. Midwest prairie and Amazon rainforest. We also compared the resistome of these two major ecosystem types with that of an undisturbed Arctic tundra to provide a tropic to polar latitudinal gradient, and all with well-characterized metadata.

We addressed the following objectives: (i) what are the types and quantities of ARGs in soils of three climate regions (tundra, temperate, and tropical), (ii) what are the impacts of major land use changes on the ARG profiles, and (iii) define which ARGs are common, perhaps universal background, and within the ARGs, which are most frequent, do they commonly co-occur and their relevance to risk. The results of this study should improve our understanding of the background level and classes of soil ARGs and allow for better evaluation of the public health risk of ARGs in the environment.

## Materials and methods

### Experimental design and site description

The metagenomic sequence data used herein were from our previous studies, and used to assess the impact of land use change [13, 28] and global warming [29, 30] on soil microbial communities. The sample site locations of the 26 soil metagenomes used in this study are depicted in Additional file 1: Fig. S1. The Alaskan soils were sampled at 15–25 cm depth (active layer; above permafrost boundary) at a moist acidic tundra area in Interior Alaska near Denali National Park (63° 52′ 59″ N, 149° 13′ 32″ W) in May 2010. The Oklahoma soils were collected by soil core 0–15 cm deep from a tallgrass prairie located at the Great Plain Apiaries in McClain County, Oklahoma, United States (34° 59′ N, 97° 31′ W) in 2011, 2012, and 2013. The site was abandoned from field cropping 40 years ago with light grazing until 2008. Three additional tallgrass prairie ecosystem sites were sampled in the summer of 2009 in the US Midwest from a 750 km transect from Kansas through Iowa to Wisconsin. At each site soil was sampled from a native (never tilled) prairie and an adjacent cultivated (> 100 years) soil matched for soil edaphic traits and landform. The native prairie soils had been grazed by cattle. All cultivated soils had received manure application. The Amazon soils were sampled at the Amazon Rainforest Microbial Observatory site (10° 10′ 05″ S and 62° 49′ 27″ W) in April 2009. Five soil cores of 0–10 cm deep were collected from a primary rainforest and an adjacent 38-year-old converted pasture. The Amazon rainforest soils were never grazed, while the pasture had been continuously used for beef cattle grazing since conversion. Detailed information about the climate, vegetation, soil type, and chemistry at each sampling site were described previously [11, 28, 30, 31].

### Shotgun sequencing

Sequencing of Alaska, Oklahoma, and Amazon soils was performed on the Illumina HiSeq 2000 platform with 150-bp paired-end strategy by the Joint Genome Institute (JGI). Illumina GAIIx paired-end sequencing augmented with some 454 GS FLX sequencing for the Iowa, Kansas, and Wisconsin soils. The Alaskan soils were also sequenced by Illumina using paired ends but by Los Alamos National Laboratory.

### Identification of ARGs in shotgun data

Adapter reads in the sequence data were removed, the remaining reads were filtered to discard bases with a quality score < 20 and length < 50 base pair (bp) by SolexaQA v.3.1.7.1 [32]. To eliminate the differences caused by variations in the sequencing depth among samples, 200 million reads were randomly picked from each sample. The retrieved sequences were then used to search for ARGs following ARGs-OAP v2.0 pipeline as described by Yin et al. [27]. The SARG database identifies ARG types (antibiotic class) and within that class subtypes (e.g., a subtype having > 80% identical aligned bases based on HMM model). Diversity data are derived from the number of subtypes. The parameters used for ARG identification were alignment length cut-off of 75 nucleotides, alignment *e* value cut-off of 10^−7^, and alignment identity of 80%. The abundances of ARGs were normalized by 16S rRNA gene expressed as: Abundance = $\sum \limits_1^n\frac{\mathrm{N}\mathrm{ARG}-\mathrm{like}\kern0.17em \mathrm{sequence}\times \mathrm{Lreads}/\mathrm{L}\mathrm{ARG}\kern0.17em \mathrm{reference}}{\mathrm{N}16\mathrm{S}\kern0.24em \mathrm{sequence}\times \mathrm{Lreads}/\mathrm{L}16\mathrm{S}\kern0.24em \mathrm{sequence}}$ [26]. Analysis of metagenome data of the Earth Microbiome Project shows that the 16S rRNA gene copy number of all soils is very narrow, with a mean of 2.2 16S rRNA copies per cell [33].

### Classification of ARG hosts in de novo assembly

De novo assembly was done with MEGAHIT and default parameters. The statistics of assemblies is in Additional file 1: Table S1. ARG-carrying contigs were identified with SARG database and the cutoffs in ARGs-OAP pipeline. The ARG-carrying reads were then classified taxonomically using a contig classification tool, CAT [34].

### Identification of clinical ARGs

We define clinical ARGs as those found in human pathogens. Protein sequences of human disease associated bacterial genomes were collected from the Pathosystems Resource Integration Center (PATRIC) [35]. The collected protein sequences were searched against SARG database, hits with identity ≥ 80%, and alignment coverage (alignment length/reference ARG length) ≥ 80% were kept as clinical ARGs. Reads annotated as ARGs by SARG database were extracted from metagenomes of Amazon rainforest and pasture soils, and they were evaluated by BLAST against the protein sequences of clinical ARGs. The hits with identity ≥ 80%, alignment length ≥ 75 bp nucleotides, and *e* value ≤ 10^−7^ were regarded as clinical ARGs. The abundance of a clinical ARG was calculated with the same formula to calculate ARG abundance in ARGs-OAP v2.0 pipeline.

To comprehensively understand the homology between ARGs in soil and ARGs found in clinical settings, 12 ARGs in Amazon data sets were assembled with Xander, a target gene assembler [36, 37]. The 12 ARGs were chosen because (1) they were found in human pathogens in PATRIC database; (2) they were detected in Amazon pasture and rainforest soils at sufficient abundance so that assembly of intact/near-intact genes was possible; and (3) hidden Markov models (HMMs) are available. The seed sequences and HMMs were obtained from SARG database [27]. Assembled protein contigs of ≥ 100 amino acids were kept to evaluate sequence similarity with clinical ARG subtypes by BLASTP.

### Soil bacterial community

Bacterial taxonomic classification and abundance quantification were analyzed following the SSUsearch pipeline [38]. Briefly, a 16S rRNA gene HMM was used to search against metagenomic data and the hits were annotated with SILVA database. The sequences aligned to a part of 16S V4 variable region (577–657) and with lengths greater than 70 bp were extracted. The extracted 16S rRNA gene sequences were then clustered to estimate OTU number at 95% identity.

### Statistical analyses

Only ARGs detected with more than two reads across all samples were retained for further analyses. Bray-Curtis distance-based principal coordinates analysis (PCoA) was performed to estimate the variance of resistome profiles. Procrustes analysis was used to assess the relationship between resistome profile and bacterial community structure, 9999 permutations were used to test the significance. ARGs detected in at least five samples with a maximum read number > 5 in at least one sample were kept for ANOVA and network analyses. The differences in ARG abundances across three soils were tested by ANOVA analysis (least significant difference, *p* < 0.05). Spearman’s correlation coefficients were calculated based on the read number among soils. Network analysis was performed in Cytoscape to identify ARG clusters; only ARGs with significant (least significant difference, *p* < 0.01) and strong Spearman coefficients (> 0.9) were used. The ANOVA and Spearman correlation analyses were conducted using SPSS 23.0. PCoA, Adonis and Procrustes analysis were done with R3.5.1.

## Results

### Detected ARGs and regulatory genes

**Fig. 1:**
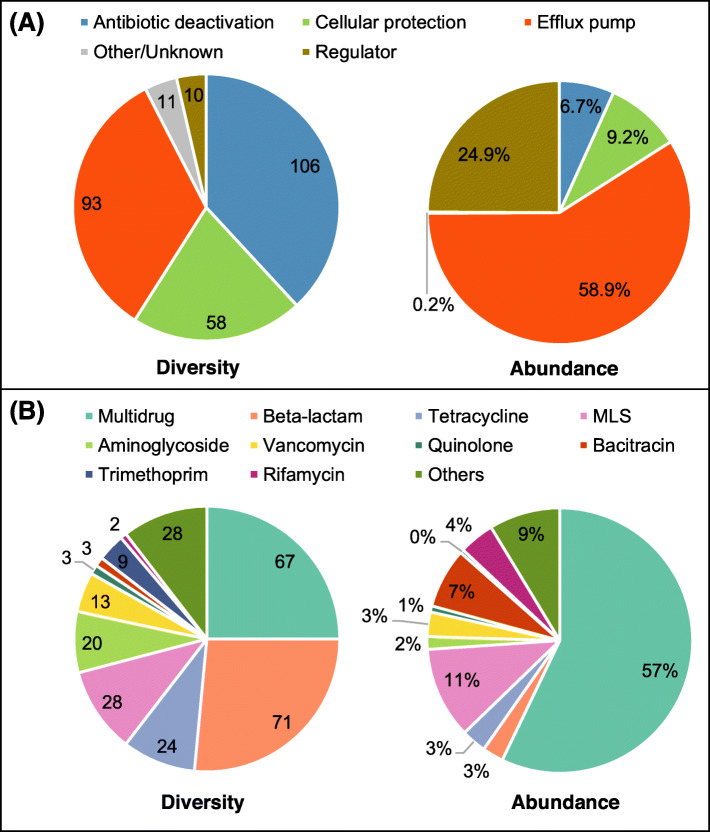
Composition of ARGs and regulator genes in 26 soil metagenomes. **a** Resistance mechanism. **b** Antibiotic classes

A total of 268 ARG subtypes potentially conferring resistance to 21 classes of antibiotics were detected in the soils, with most of them belonging to antibiotic deactivation (106 ARGs) and efflux pump (93 ARGs) mechanisms (Fig. 1). More than 58% of the resistome abundance was contributed by efflux pump genes, while only 16% was from the deactivation and cellular protection classes. Ten regulatory genes (*mtrR*, *gadX*, *tetR*, *mexT*, cAMP-regulatory protein, *arlR*, *ompR*, *vanS*, *cpxR*, *vanR*) were detected and they accounted for 13 to 35% of resistome abundances in the studied soils. Five regulatory genes were dominant totaling 0.014–0.141 copies per 16S rRNA gene copy and were observed in all 26 soils (Additional file 1: Fig. S2). Since regulatory genes do not directly confer resistance and they inflate the quantitation, they are not included in most of the following analyses.

Multidrug resistance genes were most abundant (57.1% of non-regulatory resistome abundance) with 67 subtypes observed, followed by macrolides-lincosamides-streptogramines (MLS) resistance genes, with 28 subtypes that comprised 11.2% of non-regulatory resistome abundance (Fig. 1b). There were 71 beta-lactam, 24 tetracycline, and 20 aminoglycoside resistance gene subtypes detected in these soils, but they on average only accounted for 2.6%, 3.0%, and 1.6% of non-regulatory resistome abundances, respectively.

### Non-regulatory ARGs shared by all soils

**Fig. 2:**
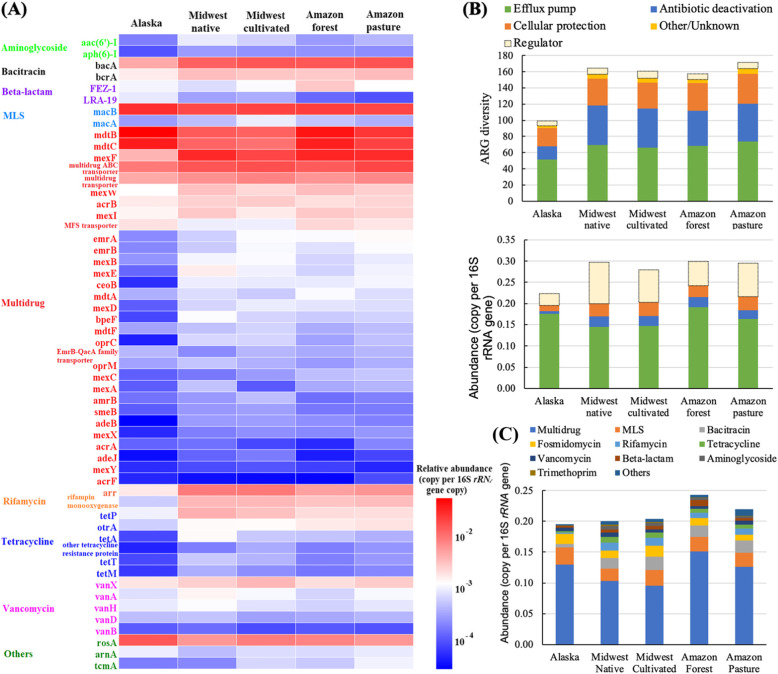
Diversity and abundance and of ARGs among soils of three ecosystems. **a** Heatmap showing abundances of 55 background ARGs in 26 soil metagenomes. **b** diversity and abundance of ARGs by different mechanisms. **c** ARG abundance of different antibiotic classes. MLS: macrolides-lincosamides-streptogramines

Fifty-five non-regulatory ARGs were shared by all 26 soils (Fig. 2a). These commonly shared ARGs accounted for 81.5 to 98.6% of non-regulatory resistome abundance across all soils, regardless of native or anthropogenic. The shared ARGs consist of nine classes of antibiotic resistances, but most of them are multidrug resistance genes (31 subtypes, 1.7 × 10^−2^–6.7 × 10^−1^ copies per 16S rRNA gene copy). There were six tetracycline resistance genes and five vancomycin resistance genes shared by all soils, and they comprised 1.0 × 10^−3^–1.2 × 10^−2^ of resistome abundance. Only two beta-lactam resistance genes were found in all soils, with abundance of 4.6 × 10^−4^–3.8 × 10^−3^ copies per 16S rRNA gene copy. The concentrations of shared aminoglycoside and trimethoprim resistance genes differed considerably across the three ecosystems, ranging from 9.4 × 10^−5^ to 2.0 × 10^−3^ copies per 16S rRNA gene copy. Efflux pump was the dominant mechanism of these 55 shared ARGs, contributing 80.6% of the total shared ARG abundance.

### ARGs in native soils

**Fig. 3:**
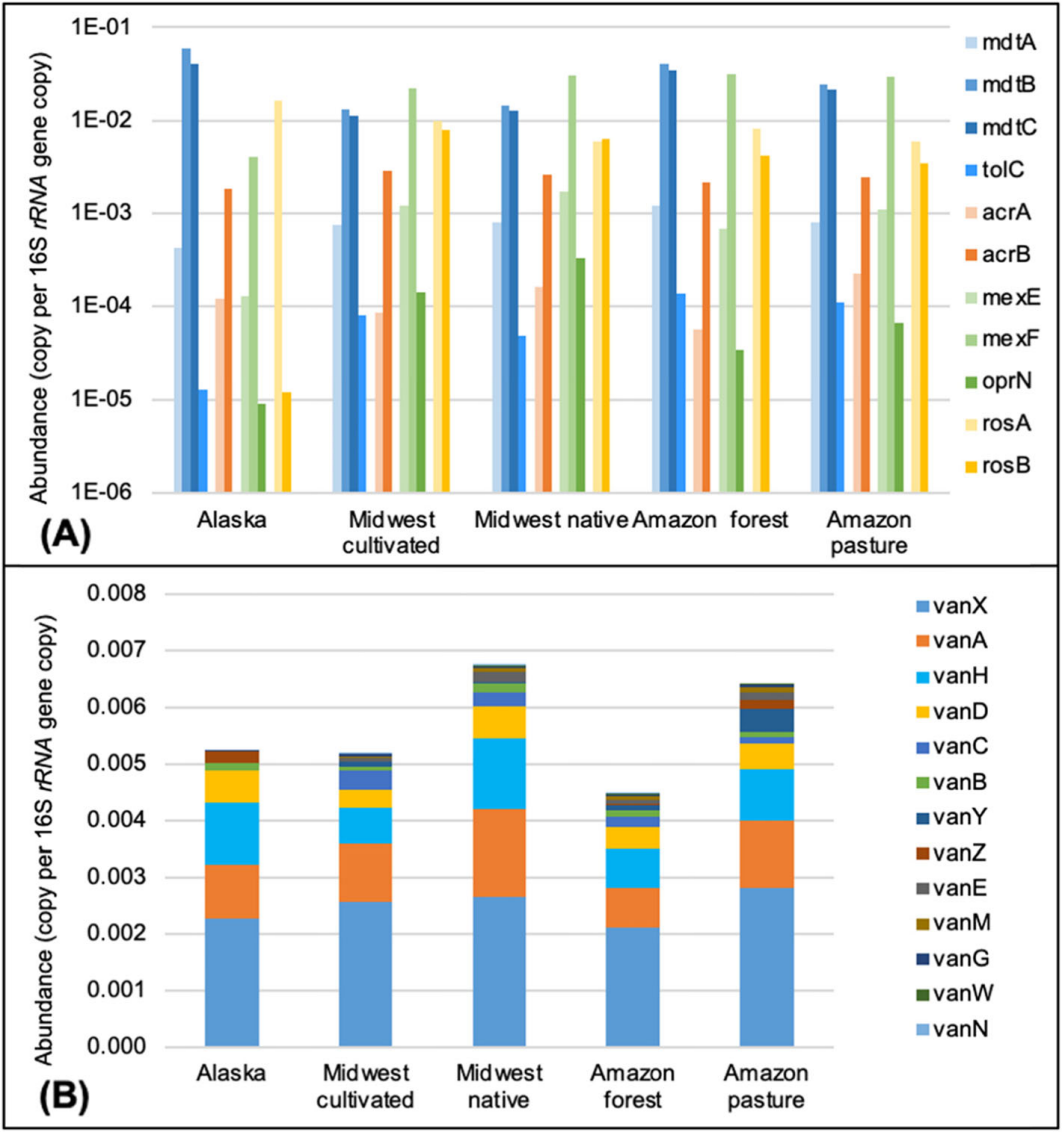
Abundance of selected ARGs among soils of the three ecosystems. **a** Efflux pump complex. **b** vancomycin resistance genes

A total of 242 ARG subtypes were observed in the native soils, covering all detected classes of antibiotic resistance (Fig. 2b). There were 144, 191, and 215 ARG subtypes with resistome abundances of 0.195, 0.201, and 0.243 copies per 16S rRNA gene detected in Alaskan tundra soil, Midwestern US native prairie soils and Amazon rainforest soils, respectively. There were no significant differences (*p* > 0.05) in ARG diversity nor resistome abundance between native and anthropogenic soils. Much of the resistome abundance was contributed by gene components of efflux pump complexes, such as *mdtABC-tolC*, *acrAB*, *mexEF-oprN*, and *rosAB* (Fig. 3a). Thirteen subtypes of vancomycin resistance genes were detected in native soils, and no significant difference was observed (*p* = 0.07) between native and anthropogenic soils (Fig. 3b).

### Soil resistome at different geographical locations

**Fig. 4:**
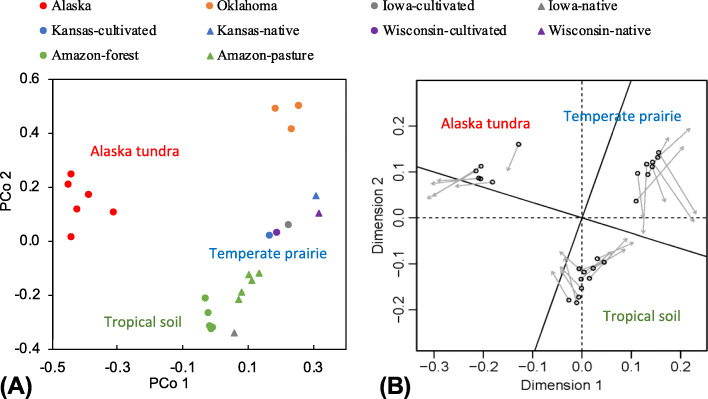
Soil resistome profiles. **a** PCoA analysis showing profiles of soil resistomes in three ecosystems. Circles represent native soils, and triangles represent anthropogenic soils. **b** Procrustes analysis of bacterial community and resistome profile. Data point shows the position of a soil sample in the ordination based on resistome profile, and arrow points to its position in the transformed ordination based on bacterial community structure

PCoA analysis clearly demonstrates that soil resistome profiles grouped by geographic location (Fig. 4a). ARG diversity in the tundra was significantly lower (*p* < 0.01) than that of temperate and tropical areas (Fig. 2b), while no significant difference (*p* = 0.91) was found between temperate and tropical areas. All ARGs detected in Alaskan soils were found in Midwest America and Amazon soils (Additional file 1: Fig. S3). One-hundred and forty ARGs were observed in both temperate and tropical soils, but not in tundra. The temperate and tropical soils shared 87.6% of ARGs, while 27 ARGs were only found in one or the other. The tropical soils had the highest resistome abundance, but no statistical difference (*p* > 0.05) was found among the three areas (Fig. 2c). Procrustes analysis showed that the ARG profile was significantly correlated with the bacterial community structure (sum of squares *M*^2^ = 0.183, *r* = 0.904, *p* < 0.01) (Fig. 4b).

### Effect of cultivated agriculture on soil resistome

There were no observed significant differences (*p* > 0.05) in either diversity or total ARG abundance between the US Midwest native and the long-term cultivated soils (Fig. 2b). The resistome profiles were very similar in the cultivated soils from the three sampling sites (Fig. 4a). The vancomycin resistance genes in native soils were higher than in cultivated soils, but the difference was not significant (*p* = 0.56) (Fig. 3b).

### Changes of soil resistome during conversion of the Amazon rainforest to pasture

Conversion of Amazon rainforest to pasture significantly (Adonis test, *R* = 0.148, *p* < 0.05) altered the soil resistome profile (Fig. 4). The conversion to pasture led to an increase of 23 ARG subtypes in pasture soils (Additional file 1: Fig. S4). All exclusive ARGs (in rainforest or pasture soils), except *dfrB2*, had a relatively low abundance, ranging from 1.8 × 10^−5^–3.1 × 10^−4^ copies per 16S rRNA gene. The non-regulatory resistome abundance in Amazon pasture soil was 11.8% lower than that in the native rainforest soil, although it was not statistically different (*p* = 0.06).

### Identification of ARG hosts in de novo assemblies

**Fig. 5:**
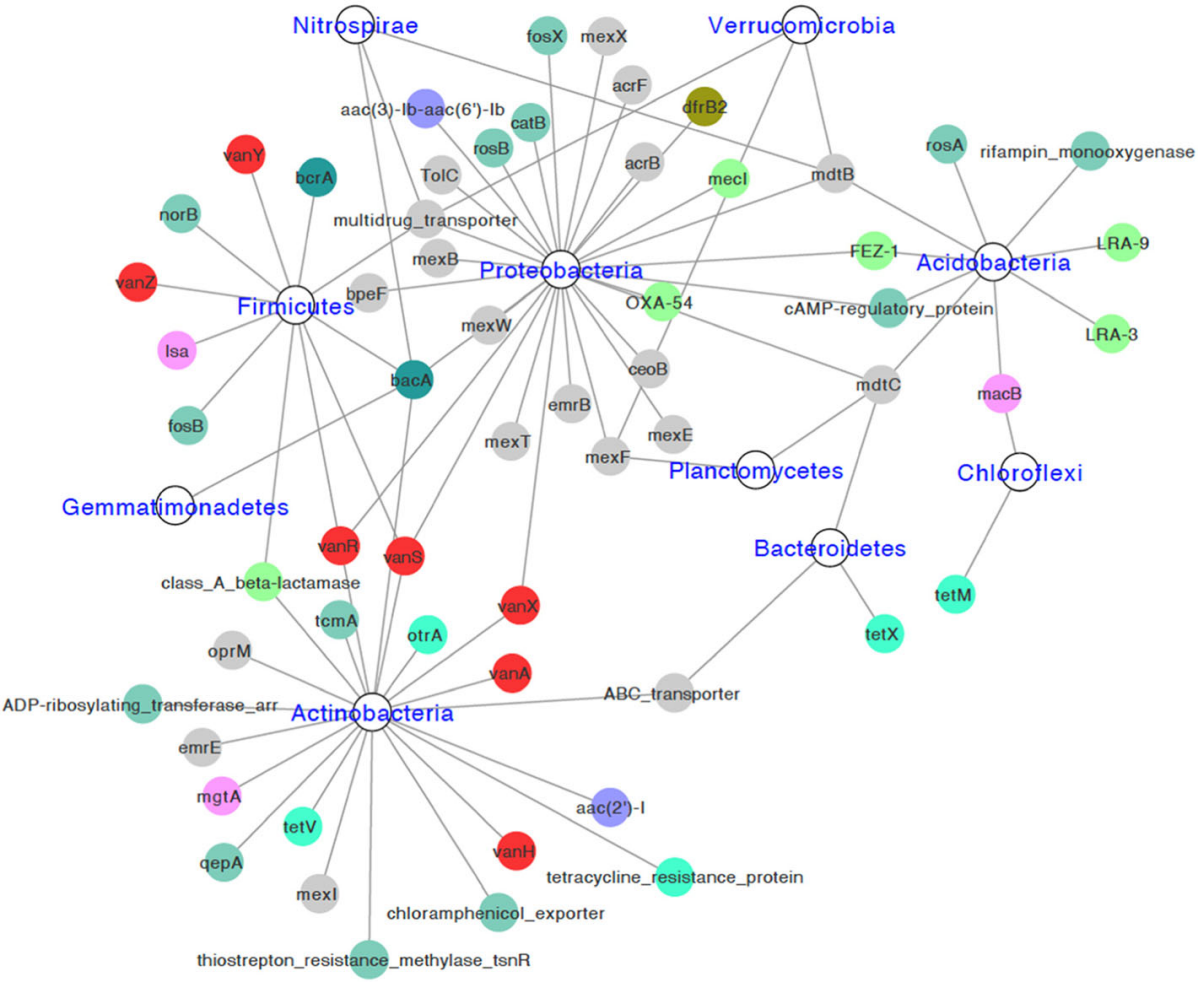
Network showing identified hosts of ARGs at phylum level. Different colors represent different classes of ARGs

We identified 77–208 ARG-containing contigs in de novo assemblies of studied soils, with N50 lengths from 414 to 14,414 bp (Additional file 1: Table S1). Approximately 81.5% of contigs had ARG coverage less than 30%, only four contigs contained intact ARGs (one *catB*, one *dfrB2* and three cAMP-regulatory proteins) (Additional file 1: Fig. S5). Hosts of 59 ARG subtypes were classified to phylum, among them 20 were multidrug resistance genes, 7 were vancomycin resistance genes, 6 were beta-lactam resistance genes, and 5 were tetracycline resistance genes (Fig. 5). Proteobacteria, Actinobacteria, Firmicutes, and Acidobacteria were primary ARG hosts, they carried 28, 21, 11, and 9 ARGs respectively. *bacA*, *mdtB*, *mdtC*, and multidrug_transporter gene had most wide host phyla (≥ 4). Regulatory genes v*anR* and *vanS* were classified to Proteobacteria, Firmicutes and Actinobacteria.

### Detection of clinical ARGs in soil resistome

**Fig. 6:**
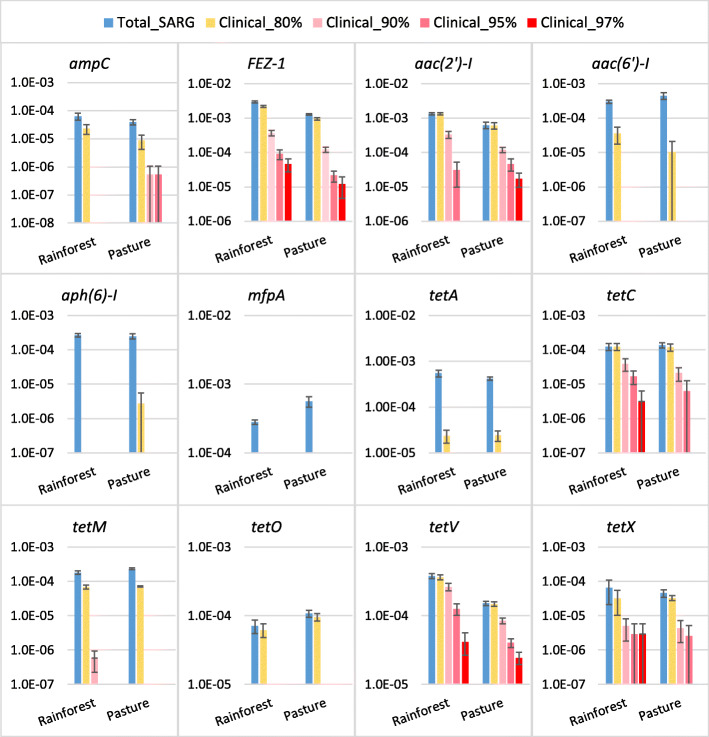
Abundance of 12 clinical ARGs in Amazon rainforest and pasture soils. The blue column represents the ARG abundance quantified with SARG database. The yellow and red columns are abundances of clinical ARGs at 80%, 90%, 95%, and 97% amino acid identities. Bars are standard errors

Since most of the above assembled ARGs are multidrug and regulatory genes and are not recognized in the PATRIC database, we targeted the 12 that were detected and clinically important to evaluate at the sequence level. These include two beta-lactam (*ampC* and *FEZ-1*), one quinolone (*mfpA*), three aminoglycoside (*aac*(*2’*)*-I*, *aac*(*6’*)*-I*, and *aph*(*6’*)*-I*), and six tetracycline resistance genes in Amazon rainforest and pasture soils (Fig. 6). Clinical-similar reads were detected in all 12 ARGs except *mfpA* at amino acid identity cut-off of 80%. Among them, more than 74% of *FEZ-1*, *aac*(*2’*)*-I*, *tetC*, *tetO*, and *tetV* reads had this level of amino acid identity to their clinical types. Only six ARGs were recovered with 90% sequence similarity. Highly similar (97% identity) clinical ARG reads were detected for *FEZ-1*, *tetC*, and *tetX*, but they only account for 0.9–4.4% of their environmental (clinical and non-clinical) abundance and none had sequences identical to those ARGs in clinical pathogens. *tetV* was the most abundant clinical ARG, with abundance of 4.1 × 10^−4^ and 2.4 × 10^−4^ copies per 16S rRNA gene in Amazon rainforest and pasture soils, respectively. No statistically significant difference (*p* > 0.05) was found in abundance of clinical ARGs at all chosen identity levels between Amazon rainforest and pasture soils. Clinical-similar *ampC* was only detected in Amazon pasture soil at identity level ≥ 90% but its abundance was as low as 5.3 × 10^−7^ copies per 16S rRNA gene.

Only three of the 12 selected ARGs could be assembled using the more sensitive target gene assembler, namely *ampC*, *tetM*, and *tetO*. Assembled *ampC* was only found in Amazon forest soils, and it shared 46–56% of amino acid similarity with clinical *ampC*. Assembled *tetM* and *tetO* shared 62–71% and 69–74% of similarity to their clinical types found in human pathogens. Consistent to the results by SARG pipeline, no significant differences were found in assembled *tetM* and *tetO* abundances between Amazon forest and pasture soils.

### Identification of ARG clusters in soils

Network analysis was performed to identify ARG clusters present in all soils (Additional file 1: Fig. S6). We found wide co-occurrence of multiple genes despite their distant geographic locations, various ecosystems, and with or without anthropogenic activities. A total of eight types of ARG clusters were observed, comprised of 67 ARG subtypes for 12 classes of antibiotics. Most of the co-occurred ARGs are multidrug resistance genes (27 subtypes), followed by tetracycline resistance genes (9 subtypes) and beta-lactamase resistance genes (8 subtypes). Four aminoglycoside resistance genes were found in ARG clusters and they had the highest average linkage with 4.8 to other ARGs. The largest ARG cluster was comprised of 25 ARGs, where *ThinB* and *aac*(*3*)-*IV* were the hub genes connecting 10 and 9 other ARGs, respectively.

## Discussion

The high diversity and abundance of the soil resistome in Alaskan tundra, temperate prairie, and tropical ecosystems support the view that ARGs are naturally ubiquitous, and in widely different terrestrial ecosystems. The ARGs detected in native soils include those that can potentially confer resistance to all major antibiotics used to treat humans and animals, such as beta-lactams (*LRA*, *PER*, *TEM*, and *OXA* genes), macrolides and lincosamides (*erm* genes), quinolones (*qepA*), aminoglycosides (*aac* and *aph* genes), and tetracyclines (*tet* genes). It is not surprising that ARGs naturally exist in native soils [3] because many antibiotics are produced by soil microorganisms, and indeed were the original source of pharmaceutical products [39]. In accord with our observation, previous studies also identified divergent beta-lactamase resistance genes and a novel chloramphenicol resistance gene from undisturbed Alaskan soil [40, 41]. Vancomycin is regarded as the last line of defense against MRSA strains, but thirteen subtypes of vancomycin resistance genes were detected in these native soils, including *vanH*, *vanA*, and *vanX* which are found in clinical pathogens *Staphylococcus aureus* and vancomycin-resistant enterococci [42]. Similarly, D’Costa et al. detected the three vancomycin genes in 30,000-year-old permafrost sediments, and further analyses confirmed the similarity in structure and function between the ancient *vanA* and their modern variants [43]. Our ARG host analysis showed that *vanH*, *vanA*, and *vanX* were harbored by Actinobacteria which are vancomycin producing bacteria [44].

We identified 55 structural ARGs and 5 regulatory ARGs that were shared by all soils regardless of ecosystem type and geography, and hence are candidate common or “background” soil ARGs. These ARGs were also found across various terrestrial ecosystems in previous studies. Thirty of our background ARGs were detected in paddy soils [4]; ten were found in dryland (peanut) soils [9]; at least sixteen were observed in greenhouse soils [45] and ten were found in Antarctic soils [5]. Most of the shared ARGs are multidrug resistance genes with efflux pump as the dominant mechanism. For example, 11 of the background ARGs are involved in Mex-Opr efflux pump systems, and they are known to play a prominent role in the multidrug resistance of gram-negative bacteria [46, 47]. The AcrAB efflux pump plays a physiologic role of pumping out bile acids, fatty acids, and various toxic compounds [48, 49]. Thus, we argue that these commonly shared ARGs should be considered as a separate category, generally of low risk, when evaluating ARG risk in soil environments. However, this does not mean that background ARGs are risk free, since some of them have been found in plasmids and can be enriched with anthropogenic activity. For example, *macB* can be easily acquired by mobile elements, and thus spread macrolide resistance [50]. Background ARGs *acrA*, *vanC*, and *mexF* were found significantly enriched by the application of sewage sludge and chicken manure to soil [51]. These results imply that compared to the abundance of ARGs, assessment of ARG mobility may be more important for ARG risk evaluation since ARG transfer into pathogens is a primary risk factor.

The soil resistome profile had a significant geographic pattern, which was greater than land use change. The significant correlation (*p* < 0.01, *R*^2^ = 0.795) between ARG diversity and bacterial diversity (Additional file 1: Fig. S7A) suggests that the lower bacterial diversity may explain the lower ARG diversity in tundra. Similar to our findings, Wang et al. [5] detected a positive correlation (*R*^2^ = 0.39, *p* = 0.0001) between bacterial Shannon index and Shannon index of ARGs in Antarctic soils. In contrast, no significant correlation (*R*^2^ = 0.152) was observed between ARG abundance and bacterial diversity (Additional file 1: Fig. S7b), which is inconsistent with Bahram et al. [23] who investigated the microbiome of global topsoil samples (189 sites, 7560 subsamples, 12 ecosystems) and found significant negative correlation between ARG abundance and bacterial diversity. The inconsistency could be due to the difference in soil edaphic traits, vegetation, land use history, and which genes are included. For example, Bahram et al.’s study used ARDB for ARG annotation and included regulatory genes such as *vanR* and *vanS* in analyses while all regulatory genes were excluded from our analyses. Significant correlation between the resistome profile and microbial community structure was observed, indicating that the differences in ARG profile is primarily driven by bacterial composition. This is consistent with previous studies which also found strong correlation between ARG profile and bacterial community structure in various environments [52–54]. Thus, the variation of soil resistomes at different geographical locations was probably related to the differing vegetation, climate, and edaphic factors such as pH and soil organic matter [55–57] which will select different populations (and hence the ARGs they carry) or some ARGs by their alternative function(s).

It is well known that the introduction of selective or co-selective pressure by human activities is primarily responsible for the enrichment of ARGs in soils. For example, irrigation with reclaimed water led to enrichment of 60 ARGs [8]. Long-term application of pig manure significantly enhanced the abundance of *tetL*, *tetB*(*P*), *tetO*, *tetW*, *sul1*, *ermB*, and *ermF* as compared with inorganic fertilizers [58]. However, it is not clear whether the normal agricultural activities such as crop production affect the soil resistome. In this study, no significant change was observed in either ARG diversity or resistome abundance after long-term continuous cultivation. However, the cultivated soils from the three Midwest sites tend to have similar resistome profiles which may be due to selection for similar adaptations of the bacterial community to agronomic production. Cropping system type, fertilization, and other soil management practices are thought to be factors that can influence the soil resistome [9]. In these study sites, antibiotics and heavy metals were not used so external factors would not have provided for selection. Overall, our results suggest that standard cultivation and fertilization practices of US Midwest (primarily moldboard plow, inorganic N.P.K, and low levels of manure, e.g., cattle grazing) did not increase the public health risk of ARGs in soil. Twenty-three new ARGs emerged and both enrichment and attenuation of ARGs were observed after conversion of Amazon forest to pasture. We speculate that the grass vegetation (*Urochloa brizantha*, *Urochloa decumbens*, *Panicum maximum*) and/or cattle grazing, which includes their manures, may be responsible for the changes in ARGs by selecting different microbial populations and/or increasing their diversity [13].

Growing evidence has shown that some ARGs in pathogens are acquired from environmental bacteria through horizontal gene transfer. For example, the CTX-M extended spectrum beta-lactamase originated from chromosomal genes of an environmental genus, *Kluyvera* [59], and the clinical *vanA* has been found in environmental *Bacilli* [60]. Thus, we selected 12 ARGs which are clinically important and could transfer between bacteria [61] and assessed their sequence similarity to clinical ARGs in human pathogens. The ARGs we detected in Amazon soils are distinct from those found in human pathogens, implying that most ARGs in the natural soil resistome are not demonstrated as problematic or at least not yet entered the clinical realm. Only a few clinical-similar reads (> 90% amino acid identity) of our tested ARGs were observed, but further evaluation of target gene assemblies confirmed that most of them were aligned to conserved regions of these genes. For example, *ampC* codes clinically important cephalosporinases which confers resistance to cephalothin, cefazolin, cefoxitin, and most penicillins, but the assembled *ampC* in Amazon soils shared less than 54% of similarity with those found in human pathogens. It is noteworthy that most researchers used short-read based BLAST for ARG search, which provides a sensitive detection but will also recover non-functional pseudo genes or conserved domains. By contrast, ARG evaluation with assembled genes will miss some low abundance ARGs but should better reflect the presence, abundance and sequence similarity of potentially functional ARGs.

There are approximately one ARG in 10 cells in our soils (assuming 2.2 copies of 16S rRNA genes per cell for soil bacteria [33]). Despite of the high resistome abundance, most of ARGs cannot be well assembled by de novo assembly. This is because 80% of soil resistome abundance was contributed by 9–17 ARGs (Additional file 1: Fig. S8). About 50–69% of ARG subtypes were less abundant than 1 in 10,000 cells. We estimated the average coverage of our soil sequence data with NonPareil [62] which showed that approximately 1.6∼11.4 terabytes of sequence data are required for 95% abundance-weighted average coverage of the temperate and tropical soil communities (Additional file 1: Fig. S9). These results show the genetic complexity of the soil microbiome and hence the difficulty of assembling more than the dominant ARGs using Illumina short reads with today’s resources. As one example, we checked the coverage of *vanS* regulatory genes and found an uneven distribution by position (Additional file 1: Fig. S10). A further BLASTX of reads annotated as *vanS* against nr database demonstrated that most of the reads are HAMP domain (present in Histidine kinases, Adenylate cyclases, Methyl accepting proteins, and Phosphatases). HAMP domain is approximately 50 amino acids long and is commonly found in integral membrane proteins and two-component regulatory systems [63]. It indicates that at least part of resistome abundance from the short-read alignments may include conserved protein domains, which would lead to overestimates of ARGs.

It is noteworthy that some regulatory genes are included in the SARG database as well as the widely used CARD database [64]. It is problematic as to whether regulatory genes should be counted as ARGs since they only control expression, and not only of ARGs. For example, *vanR* and *vanS* cannot confer resistance to vancomycin, but *vanR* can promote co-transcription of *vanA*, *vanH*, and *vanX* when activated by *vanS* [65]. A high abundance of regulatory genes was detected and they differed in soils from the several ecosystems (Fig. 2b, c). We removed regulatory genes from our further analyses since the potential risk of ARGs is largely from the horizontal transfer of structural genes which code for functional proteins. In addition to the regulatory genes, some ARGs are components of a functional complex, for which an individual ARG cannot code antibiotic resistance without others. For example, many ARGs detected in our study are components of mdtABC-tolC, acrAB-tolC, and mexEF-oprN efflux complexes. Thus, the addition of ARGs belonging to a complex can inflate the total resistome abundance.

Granted, soil is an important reservoir of ARGs; it harbors background ARGs that may or may not become problematic, probably harbors ARGs not yet emerged, and can harbor clinical ARGs, most likely to have entered soil from human or animal waste disposal. We recommend that more attention be paid to ARG genes or gene sets necessary for resistance function, for their status relative to common ARG backgrounds, for linkage to mobile genetic elements, and their correspondence or linkage to host populations. Sequence similarity may or may not be indicative of potential ARG function but it is a strong indicator of whether the ARG source was from a known clinical resistance and detectable by methods targeting the clinical gene variant.

## Conclusions

Soil harbors ARGs that may or may not become problematic, and some that are yet to emerge. We show that the ARG reservoir in soil is global, huge, and exhibits significant geographic patterns. We identified 55 structural and 5 regulatory ARGs as common in all samples of these diverse ecosystems and suggest that these candidate background ARGs be considered as a separate category for health risk evaluation. Further, soil ARGs shared low sequence similarities with those commonly found in human pathogens. We recommend that more attention be paid to ARG genes or gene sets necessary for resistance function, for their status relative to common ARG backgrounds, for linkage to mobile genetic elements and their correspondence or linkage to host populations to evaluate risk.

## Supplementary Information

**Additional file 1: Table S1.** Statistics of de novo assemblies and ARG-carrying contigs. **Fig. S1.** Geographical distribution of sampling sites. **Fig. S2.** Antibiotic resistance regulatory genes in soils with *arlR*, *cpxR*, *ompR*, *vanR* and *vanS* found in all 26 soils and part of shared background. **Fig. S3.** Venn diagram showing shared ARGs among Alaska, Midwest USA, and Amazon soils. **Fig. S4.** (A) The shared and exclusive ARGs between Amazon rainforest soils and pasture soils. (B) The composition of the exclusive ARGs. **Fig. S5.** ARG coverage (length of an assembled contig divided by length of the intact ARG) on de novo assemblies. **Fig. S6.** Network analysis assessing the ARG cluster across soils from tundra, temperate prairie and tropical ecosystems. **Fig. S7.** Pearson correlation between (A) ARG diversity and bacterial diversity; (B) resistome abundance and bacterial diversity. **Fig. S8.** Rank percentage of soil resistome abundance of top 50 ARGs. **Fig. S9.** Nonpareil curves showing estimated average coverage in soil datasets. **Fig. S10.** Per base coverage of vanS regulatory gene in 26 soils.

## Data Availability

All sequence data used in the study is available at European Nucleotide Archive (no. PRJEB10725) and JGI (Project Ids 1077701–1077706 and 1080879–1080888).
