# Supplementary material for: Metagenomic analysis reveals the shared and distinct features of the soil resistome across tundra, temperate prairie, and tropical ecosystems
Source: Microbiome. 2021 May 14;9:108. doi: 10.1186/s40168-021-01047-4 (PMC8122544; doi:10.1186/s40168-021-01047-4)
Supplement: Supplementary file 2 — Additional file 1: Table S1. Statistics of de novo assemblies and ARG-carrying contigs. Fig. S1. Geographical distribution of sampling sites. Fig. S2. Antibiotic resistance regulatory genes in soils with arlR, cpxR, ompR, vanR and vanS found in all 26 soils and part of shared background. Fig. S3. Venn diagram showing shared ARGs among Alaska, Midwest USA, and Amazon soils. Fig. S4. (A) The shared and exclusive ARGs between Amazon rainforest soils and pasture soils. (B) The composition of the exclusive ARGs. Fig. S5. ARG coverage (length of an assembled contig divided by length of the intact ARG) on de novo assemblies. Fig. S6. Network analysis assessing the ARG cluster across soils from tundra, temperate prairie and tropical ecosystems. Fig. S7. Pearson correlation between (A) ARG diversity and bacterial diversity; (B) resistome abundance and bacterial diversity. Fig. S8. Rank percentage of soil resistome abundance of top 50 ARGs. Fig. S9. Nonpareil curves showing estimated average coverage in soil datasets. Fig. S10. Per base coverage of vanS regulatory gene in 26 soils. [file 40168_2021_1047_MOESM2_ESM.docx]

**Additional File**

**Title:** Metagenomic analysis reveals the shared and distinct features of the soil resistome across tundra, temperate prairie and tropical ecosystems

**Authors:** Xun Qian^a,b^, Santosh Gunturu^b^, Jiarong Guo^b^, Benli Chai^b^, James R. Cole^b^, Jie Gu^a,c#^, James M. Tiedje^a,b#^

**Affiliations and addresses**: ^a^ Interdisciplinary Research Center for Soil Microbial Ecology and Land Sustainable Productivity in Dry Areas, Northwest A&F University, Yangling, Shaanxi 712100, China; ^b^ Center for Microbial Ecology, Michigan State University, East Lansing, MI 48824, USA; ^c^ College of Natural Resources and Environment, Northwest A&F University, Yangling, Shaanxi 712100, China

**Table S1** Statistics of de novo assemblies and ARG-carrying contigs

|  | **Number of contigs** | **Contig N50 length (bp)** | **Contigs maximum length (bp)** | **Number of ARG carrying contigs** | **ARG-carrying contig N50 length (bp)** | **ARG-carrying contigs maximum** | **Number of classified ARG carrying contigs** |
| --- | --- | --- | --- | --- | --- | --- | --- |
| Alaska | 5,639,798 | 639 | 91,887 | 77 | 600 | 3760 | 48 |
| Amazon forest | 6,947,479 | 464 | 48,986 | 103 | 414 | 1,751 | 72 |
| Amazon pasture | 6,127,175 | 464 | 136,435 | 169 | 483 | 2,067 | 129 |
| Oklahoma | 4,918,793 | 424 | 343,378 | 208 | 14,414 | 56,021 | 134 |

**Figures**


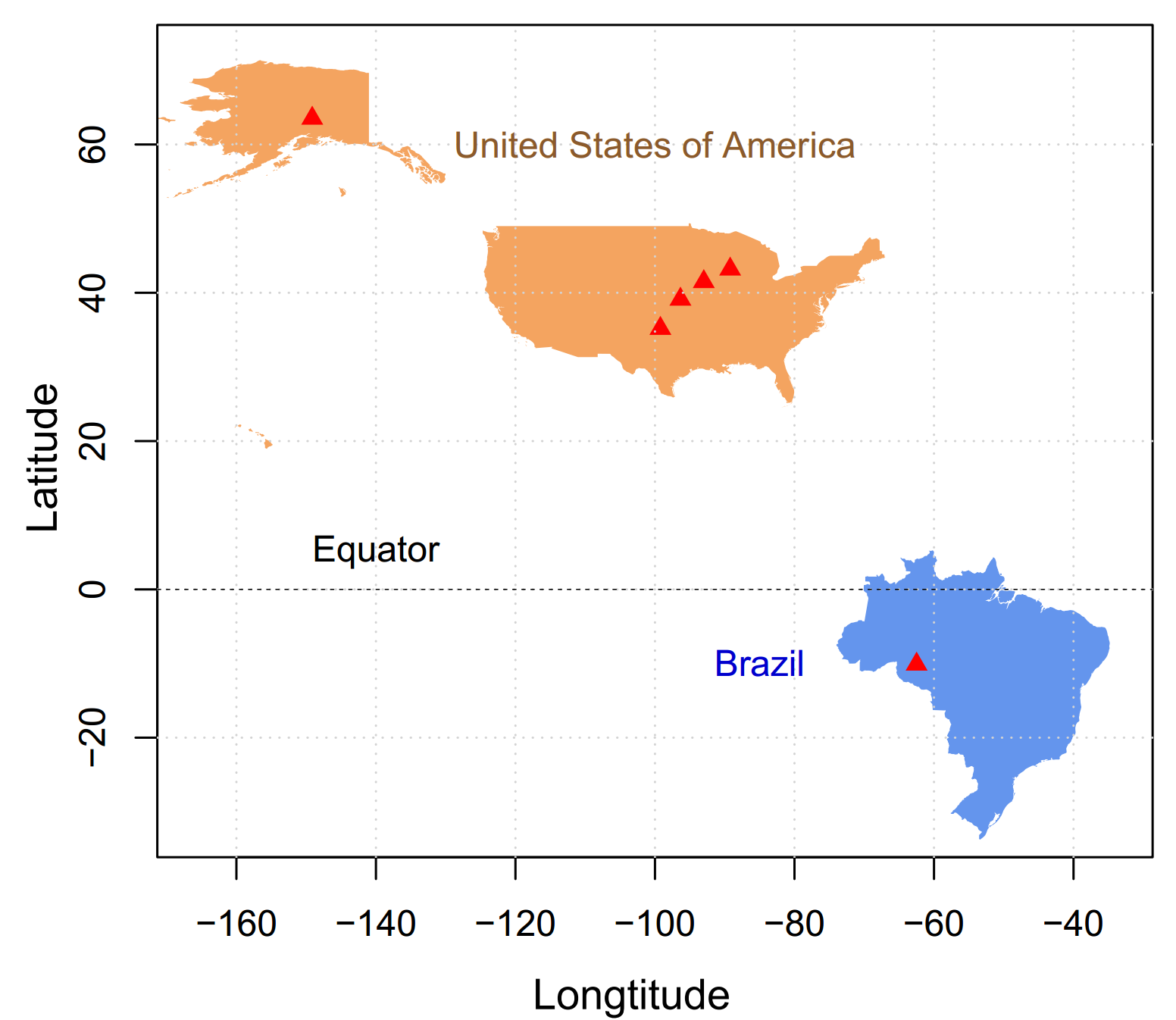


Fig. S1 Geographical distribution of sampling sites.

Fig. S2 Antibiotic resistance regulatory genes in soil with *arlR*, *cpxR*, *ompR*, *vanR* and *vanS* found in all 26 soils and part of shared background.

Fig. S3 Venn diagram showing shared ARGs among Alaska, Midwest USA, and Amazon soils.

Fig. S4 (A) The shared and exclusive ARGs between Amazon rainforest soils and pasture soils. (B) The composition of the exclusive ARGs.


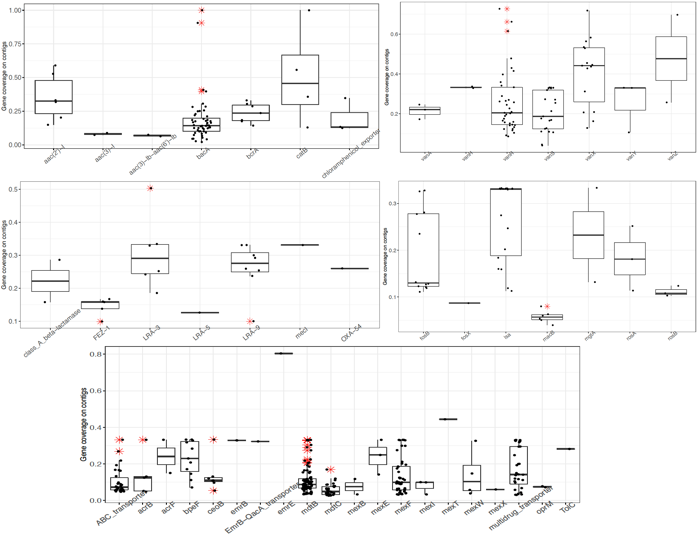


Fig. S5 ARG coverage (length of an assembled contig divided by length of the intact ARG) on de novo assemblies.


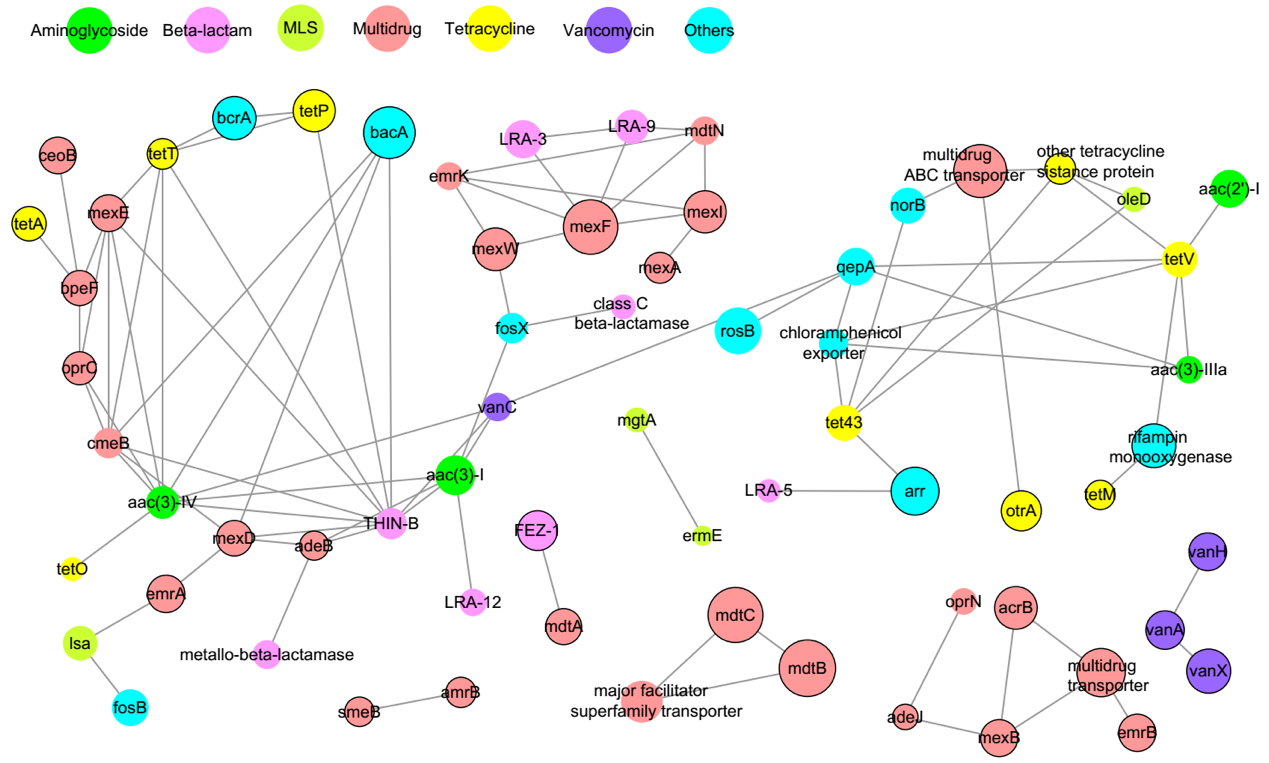


Fig. S6 Network analysis assessing the ARG clusters across soils from tundra, temperate prairie and tropical ecosystems. The lines represent significant (*p* < 0.05) and strong (R^2^ > 0.8) positive Spearman correlations. The node size is weighted according to the average abundance of each ARG. Circles enclosed with black boundary represent background ARGs. MLS: Macrolides-lincosamides-streptogramines.

Fig. S7 Pearson correlation between (A) ARG diversity and bacterial diversity; (B) resistome abundance and bacterial diversity.

Fig. S8 Rank percentage of soil resistome abundance of top 50 ARGs. The top 9 most abundant ARGs (ranked according to average proportion of resistome abundance in 26 soils) are regulatory genes *vanR, vanS* and *cpxR*, and structural genes *mdtB*, *mdtC*, *mexF*, *macB*, multidrug_ABC_transporter gene, and *bacA*.


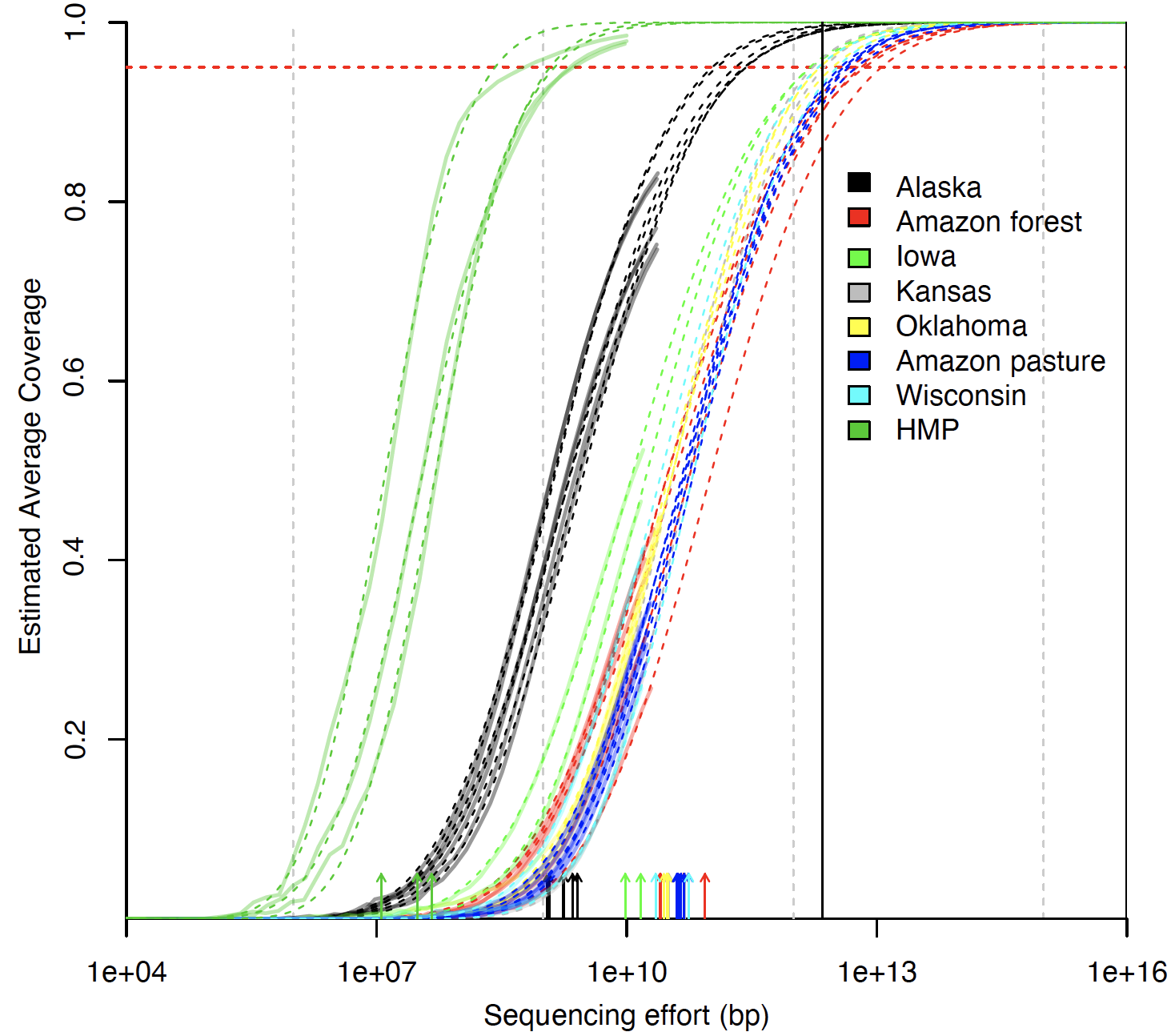


Fig S9 Nonpareil curves showing estimated average coverage in soil datasets. HMP denotes Human Microbiome Project datasets (Human Microbiome Project Consortium 2012), which is added for reference. Approximately 1.6∼11.4 terabytes of sequence data are required for 95% abundance-weighted average coverage (horizontal dashed red line) of the temperate and tropical soil communities. To acquire 60% coverage of microbial communities, the temperate and tropical soils are predicted to require 2000 times more sequence than the human gut.


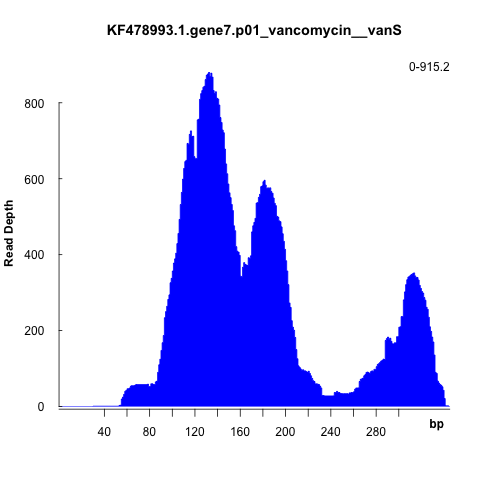


Fig. S10 Per base coverage of *vanS* gene in the 26 soils. It shows that short regions targeted by PCR or similarity search have a big influence on quantitation.

**Reference**

von Meijenfeldt, F. A. B., Arkhipova, K., Cambuy, D. D., Coutinho, F. H. and Dutilh, B. E. Robust taxonomic classification of uncharted microbial sequences and bins with CAT and BAT. *Genome Biol* 20, 217 (2019).

Rodriguez-R et al. 2018. Nonpareil 3: Fast estimation of metagenomic coverage and sequence diversity. mSystems, 3:e00039-18.

Human Microbiome Project Consortium. 2012. Structure, function and diversity of the healthy human microbiome, Nature, 486:207-214
